# Arthroscopic One‐to‐One Anchor, Double‐Row Rotator Cuff Repair

**DOI:** 10.1002/atn2.70020

**Published:** 2026-04-30

**Authors:** Sandeep Mannava, Omkar N. Prabhavalkar, Richard Lander, Brett P. Salazar, Samantha Levin, Patrick Castle

**Affiliations:** ^1^ Department of Orthopaedics and Physical Performance Divisions of Sports Medicine, Shoulder and Elbow Surgery University of Rochester Medical Center 601 Elmwood Avenue, Box 665 Rochester 14642 New York U.S.A.

## Abstract

Rotator cuff repair aims to achieve stable tendon‐to‐bone healing while restoring the anatomic footprint and minimizing tension. Although single‐row and double‐row constructs are widely used, double‐row repairs have showed biomechanical advantages, including broader footprint coverage, improved force distribution, and reduced gap formation. These benefits have translated to lower retear rates and improved functional outcomes in certain populations. This technique describes an arthroscopic one‐to‐one anchor, double‐row construct designed for small, crescent‐shaped tears. By providing multiple points of fixation with fewer implants, the construct distributes load evenly across the repair and maximizes footprint restoration.

## Graphical abstract

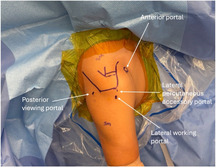

**VIDEO 1:** Arthroscopic One‐to‐One Anchor, Double‐Row Rotator Cuff Repair technique in the beach chair position. Four independent suture limbs from a 2.6 mm self‐punching RC FiberTak anchor are placed in an anterior to posterior fashion through the torn rotator cuff tendon. These sutures are then loaded into a 4.75 mm SwiveLock suture anchor, tensioned, and secured at the lateral aspect of the rotator cuff footprint. Video content can be viewed at https://doi.org/10.1002/atn2.70020.

In addressing rotator cuff tears, the primary goal of repair is to create a stable restoration of rotator cuff insertion onto bone, while minimizing tension on the repair. While open techniques were commonly used in the past, the increased prevalence and advancement of arthroscopic techniques have made this the preferred modern approach.^1^ Many repair constructs have been described to achieve the goal of rotator cuff repair, with significant debate over which specific arthroscopic technique provides the best clinical outcomes.^2^, ^3^, ^4^

Commonly, either a single‐row or double‐row repair technique has been used to address rotator cuff tears, depending on the size and pattern of the tear. While both of these techniques have proven beneficial in the treatment for smaller tears (<3 cm), double‐row techniques result in better outcomes, including lower retear rates and superior range of motion in some populations.^5^, ^6^, ^7^, ^8^, ^9^, ^10^, ^11^ Another technique that has been used is the blended, trans‐osseus equivalent, suture‐bridge technique, which builds upon the advantages of the double row repair but also ensures broader footprint coverage, less gap formation, and fewer knots to distribute load more evenly to allow for better tendon healing.^12^ Lastly, there have also been techniques developed in biologic augmentation, with the goal of limiting retear rates due to decreased healing capabilities and poorer tissue quality, especially in older patients. These interventions include biologic and synthetic scaffolds and the infusion of stem cells and growth factors to assist with the healing process.^13^, ^14^

With the advantages of the double‐row repair techniques observed, this article describes a technique of an arthroscopic one‐to‐one anchor, double‐row rotator cuff repair for the treatment of smaller‐sized tears (Video 1). Through this technique, additional points of fixation within the tendon are achieved for a more secure repair, with broader footprint coverage, compression, and distribution of load across the repair construct.

## SURGICAL TECHNIQUE

### Patient Positioning and Preparation

The patient is transferred to the operating room table and general anesthesia is induced. The patient is positioned in beach chair position with a pneumatic arm holder.^15^

### Technique

A standard posterior viewing portal is established. An anterior portal is then established lateral to the coracoid using an outside‐in technique and a cannula is placed. Once these portals have been established, a standard diagnostic arthroscopy is performed within the glenohumeral joint. Intra‐articular pathology is first addressed prior to subacromial work. The extent of intra‐articular work is beyond the scope of this manuscript but typically involves extensive debridement of chondromalacia, torn labrum, synovitis, and frayed tendon tissue. The long head of the biceps tendon and the subscapularis tendon can also be addressed intra‐articularly.

**FIGURE 1:**
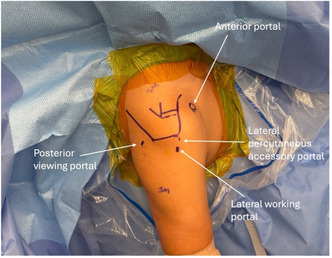
The surgical incisions of the 4 arthroscopic portals for an arthroscopic one‐to‐one rotator cuff repair are marked on a patient's right shoulder in the beach chair position. The portals are theposterior viewing portal, the anterior portal, the lateral working portal, and the lateral percutaneous accessory portal.

The camera is then moved into the subacromial space using the same posterior portal and a lateral working portal with a cannula is established roughly 2 to 4 cm lateral to the edge of the acromion. The authors’ preferred placement is around the “25‐yard” line, slightly more anterolateral (in contrast to a mid‐lateral or “50‐yard” line portal when referencing the lateral acromial edge), to allow for better access to the most common location of the small, full‐thickness rotator cuff tears (anterior and lateral).^16^ For the purposes of this manuscript, this portal will be referred to as the “lateral portal” (Figure 1). The pneumatic arm positioner can be utilized intraoperatively to ensure adequate trajectory of arthroscopic instruments to the tear and bony footprint on the greater tuberosity from the lateral portal location.

**FIGURE 2:**
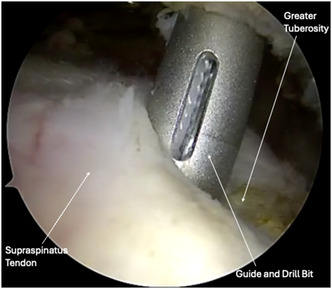
Right shoulder with the patient in the beach chair position. Viewing through the posterior portal, a drill guide is placed at the medial aspect of the footprint on the greater tuberosity, and a drill bit is used to drill a hole as the first step for anchor placement in the rotator cuff repair.

**FIGURE 3:**
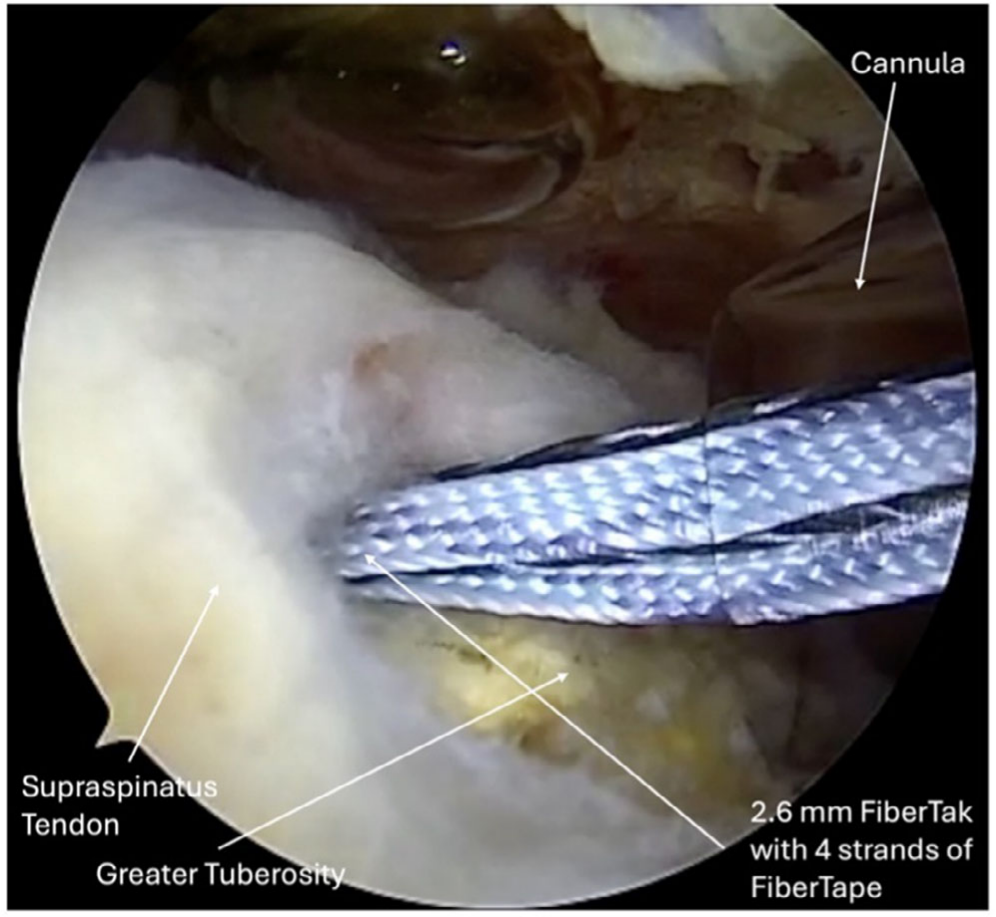
Right shoulder with the patient in the beach chair position. Viewing through the posterior portal, a 2.6‐mm FiberTak Anchor with 4 strands of FiberTape is placed at the border of the articular cartilage at the medial aspect of the rotator cuff footprint.

**FIGURE 4:**
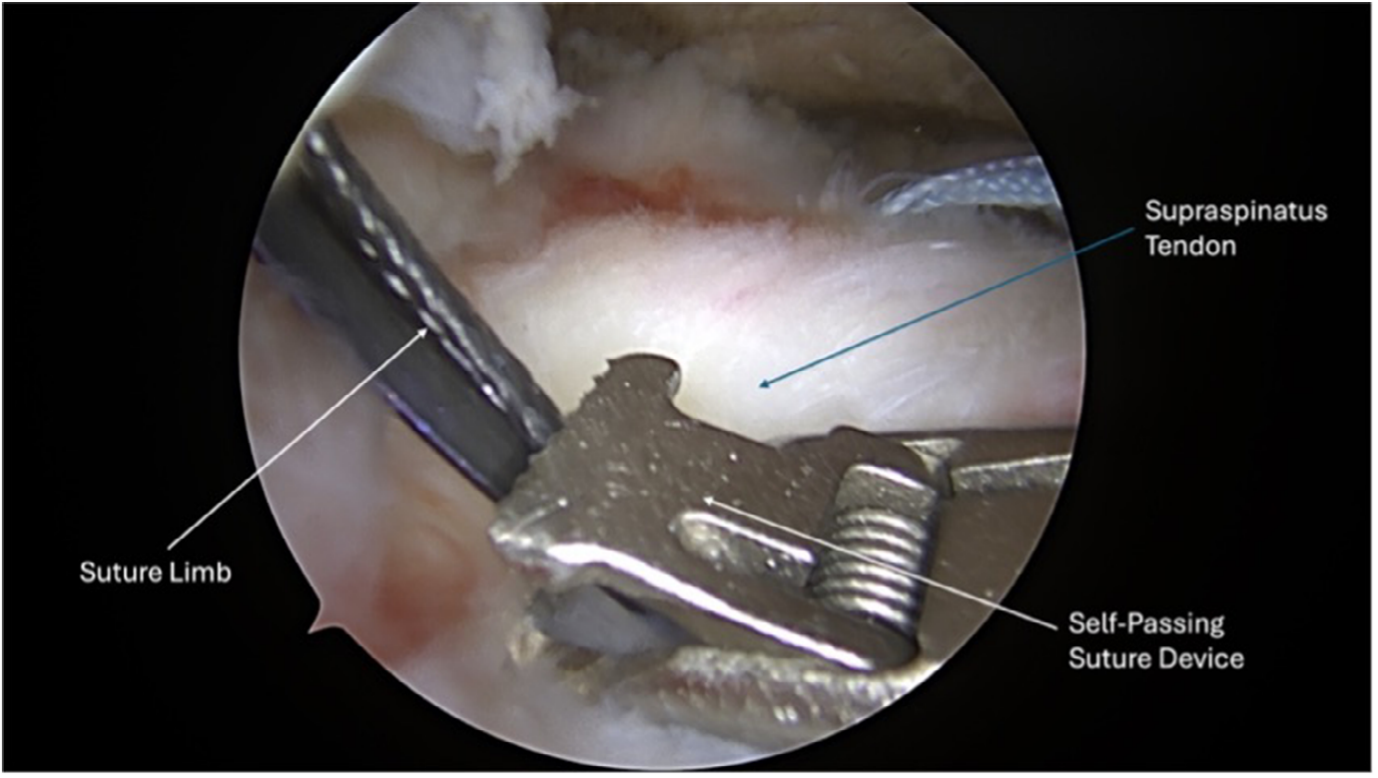
Right shoulder with the patient in the beach chair position. Viewing through the posterior portal, 1 FiberTape limb is loaded into a self‐passing suture device and placed through the torn rotator cuff tendon.

The procedure begins by preparing the greater tuberosity for repair using a radiofrequency ablation device, arthroscopic shaver, and burr. A drill guide is then placed at the medial aspect of the rotator cuff footprint, just lateral to the articular cartilage (Figure 2). This guide is placed first by needle localization and then creating a lateral percutaneous accessory portal in the more superior and typically more anterior location to the previously created lateral portal (Figure 1). Care should be taken when entering the subacromial space with the drill guide to ensure the guide does not slip, as this can cause damage to the articular cartilage or medial neurovascular structures. A drill bit is used to drill a 2.6‐mm hole for the placement of a 2.6‐mm self‐punching RC FiberTak anchor (Arthrex, Naples, FL), containing 4 strands of FiberTape (Arthrex, Naples, FL) (Figure 3). Once the anchor is placed through the guide, the swage of the sutures are cut to allow for all 4 independent suture limbs to be retrieved through a cannula (Twist‐In Cannula size 8.25 mm × 7 cm, Arthrex, Naples, FL) placed in the lateral portal location. One strand of the 4 is loaded into a self‐passing suture device (Scorpion Suture Passer, Arthrex, Naples, FL) and brought through the anterior aspect of the torn rotator cuff tendon (Figure 4). After passing it through the tendon, it is retrieved through a percutaneous lateral accessory portal for suture management, thereby parking it anterior and out of the way of the next sutures to be passed more posteriorly. A similar process is repeated for the remaining 3 sutures, placing them through the torn rotator cuff tendon in an anterior‐to‐posterior fashion, around the horn of the crescent‐shaped tear, until the sutures are equally spaced throughout the area of torn tendon. By working anterior to posterior and parking the sutures out of the percutaneous portal, each subsequent passed suture remains out of the way of viewing and there is better arthroscopic suture management, hopefully preventing suture tangling (Table 1). Care is taken to ensure equal depths of bites with each suture pass. To facilitate suture management and ease of suture identification, the colors of the sutures are alternated from dark to light when passed anterior to posterior. After all sutures are passed, they are retrieved from their parked position in the lateral percutaneous accessory portal and brought out the lateral working portal cannula.

**TABLE 1:** Pearls and Pitfalls of Arthroscopic One‐to‐One Anchor, Double‐Row Rotator Cuff Repair

Pearls	Pitfalls
Each passed suture through the crescent‐shaped rotator cuff tear should be equally spaced in the anterior‐posterior direction to maximally distribute tension and force	After tensioning each suture within the lateral row anchor but before fully seating the anchor, uncleat the sutures to prevent cut‐out of high‐tension suture through bone
During lateral anchor placement, visualize both the rotator cuff and the lateral anchor while tensioning each suture limb	Despite using a self‐punching anchor, a tap is recommended prior to anchor placement to prevent anchor breakage
When identifying the medial footprint, utilize the drill guide to lift the torn rotator cuff and visualize the tendon bone junction prior to drilling for accurate placement of the medial anchor	While utilizing the drill guide, it is recommended to apply enough pressure in order not to skive medially and damage the articular cartilage or medial neurovascular structures

**FIGURE 5:**
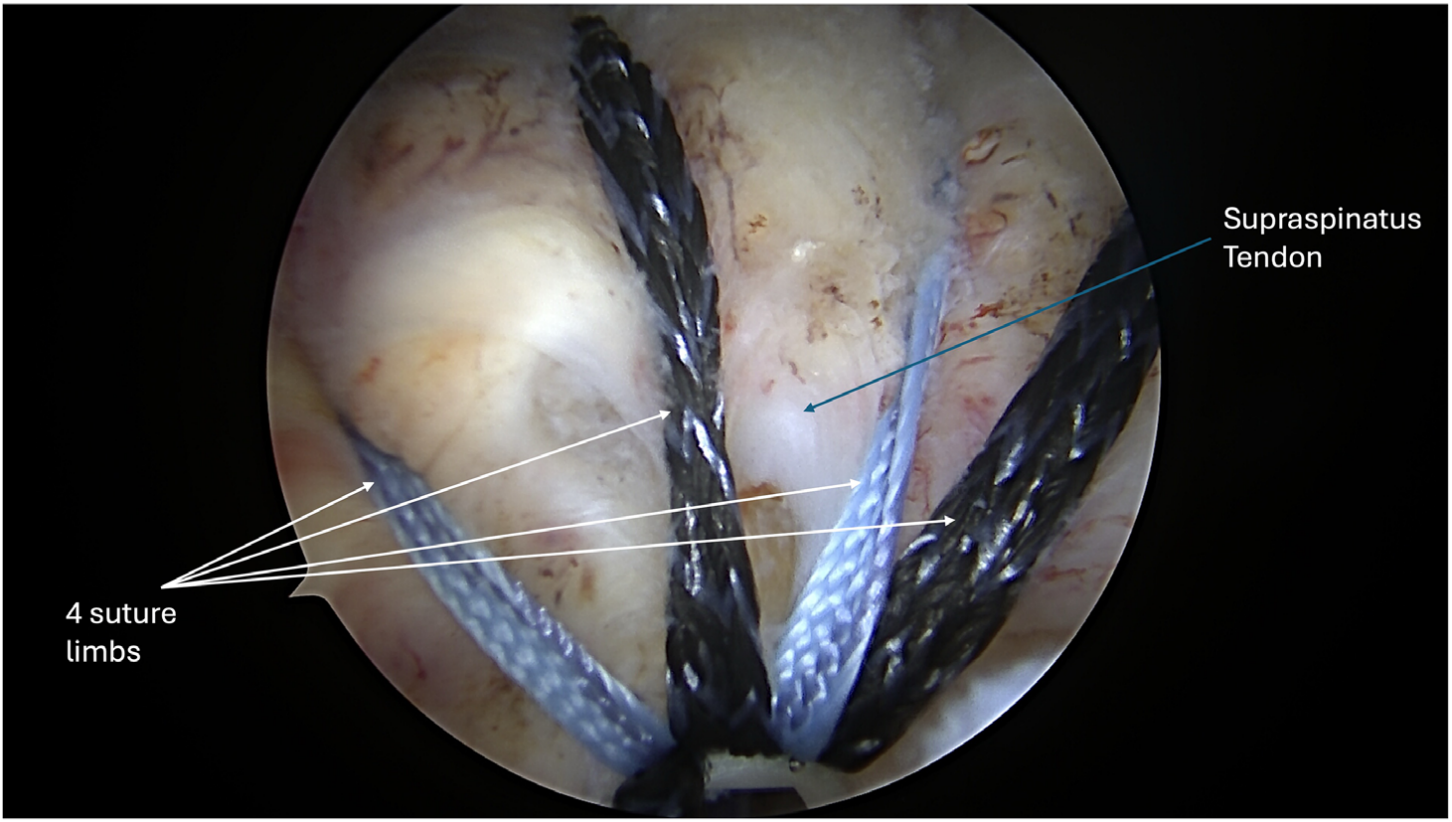
Right shoulder with patient in beach chair position. The final configuration of the arthroscopic one‐to‐one rotator cuff repair is shown through the posterior portal.

The sutures are then loaded into a 4.75‐mm SwiveLock suture anchor (Arthrex, Naples, FL). A punch is used to create a pilot hole at the lateral aspect of the rotator cuff footprint directly lateral and more distal to our initially placed medial row anchor. The punch is typically colored using a skin marker prior to placing it through the cannula to help facilitate visualization of the entry point and placement of the lateral row anchor. Despite using a self‐punching anchor, a tap is typically utilized. If the bone is particularly hard, we do recommend tapping the pathway prior to anchor placement (Table 1). The 4.75‐mm SwiveLock anchor is loaded with the 4 passed sutures. It is then placed into the lateral hole created by the punch, and each suture is individually tensioned before securing the anchor into the bone. Care should be taken to ensure that the sutures are adequately tensioned during the placement of the anchor. The sutures should be released once tension is set to ensure the high‐tensile sutures do not cut through the bone. The suture limbs are then trimmed arthroscopically, and the final configuration of the rotator cuff repair is inspected and probed for stability (Figure 5).

## DISCUSSION

Due to the tenuous nature of healing tendon to bone in rotator cuff repairs, it is important to utilize a technique that allows for a robust and stable repair with built‐in redundancy of fixation during initial fixation. There are many different factors that affect the incidence of retear rates such as tissue quality, chronicity of the tear, age of the patient, and the type of repair technique used.^17^, ^18^, ^19^

In general, techniques involving double‐row rotator cuff repair have shown superiority over single‐row techniques, likely because the double‐row construct is able to restore a larger anatomic footprint of the rotator cuff.^6^, ^9^, ^20^, ^21^, ^22^ Restoration and force distribution over the native anatomic footprint may benefit the healing process.^23^, ^24^ Double‐row repairs also provide many biomechanical advantages such as decreased gap formation and lower strain on the footprint area when compared with single‐row repairs.^20^, ^25^ The technique proposed in this article is a method of double‐row repair that allows for additional points of fixation due to the placement and spread of sutures distributed across the entire tear area in small, crescent‐shaped tears. This technique allows for distribution of tension and force over a larger area with a minimal number of anchors, allowing for a more anatomic rotator cuff repair. This may result in healing benefits as well as a more biomechanically secure repair. This technique does have some disadvantages and limitations. While this technique is effective for smaller rotator cuff tears, it is not an ideal repair technique for larger tears that would require additional anchors and suture spread. Furthermore, it depends on the patient having sufficient bone stock to perform the repair. Future studies are needed to determine whether the biomechanical advantage of this technique compared with a single‐row technique results in either faster healing or expedited rehabilitation to justify an increased cost (Table 2). Further research will be needed to compare the clinical outcomes of this repair versus other rotator cuff repair configurations.

**TABLE 2:** Advantages and Disadvantages of Arthroscopic One‐to‐One Anchor, Double‐Row Rotator Cuff Repair

Advantages	Disadvantages
Achieves relatively broad footprint coverage and improved compression across the tendon‐bone interface	Applicable to small, crescent‐shaped tears, but not ideal for larger tears
Enhances load distribution and reduces strain on the repair, improving tendon healing	Dependent on adequate bone quality; poor bone stock could compromise the fixation of the anchors
Restores anatomic footprint and minimizes gap formation compared with single‐row constructs	

## DISCLOSURES

The author (S.M.) declares the following financial interests/personal relationships, which may be considered as potential competing interests: S.M. reports a relationship with Arthrex Inc. that includes: consulting or advisory. The other authors (O.N.P., R.L., B.P.S., S.L., P.C.) declare that they have no known competing financial interests or personal relationships that could have appeared to influence the work reported in this paper.

## References

[1] Colvin AC , Egorova N , Harrison AK , Moskowitz A , Flatow EL . National trends in rotator cuff repair. J Bone Joint Surg Am. 2012;94:227‐233. 22298054 10.2106/JBJS.J.00739 PMC3262185

[2] Arroyo W , Getelman MH , Snyder SJ . Single row rotator cuff repair with triple loaded suture anchors: The SCOI row technique. Arthroscopy. 2021;37:2397‐2398. 34353551 10.1016/j.arthro.2021.06.004

[3] Shi BY , Diaz M , Binkley M , McFarland EG , Srikumaran U . Biomechanical strength of rotator cuff repairs: A systematic review and meta‐regression analysis of cadaveric studies. Am J Sports Med. 2019;47:1984‐1993. 29975549 10.1177/0363546518780928

[4] Xu B , Chen L , Zou J , Gu Y , Hao L , Peng K . The clinical effect of arthroscopic rotator cuff repair techniques: A network meta‐analysis and systematic review. Sci Rep. 2019;9:4143. 30858460 10.1038/s41598-019-40641-3 PMC6411857

[5] Gu Z , Wu S , Yang Y , Ren T , Zhang KW . Comparison of arthroscopic single‐row and double‐row repair for rotator cuff injuries with different tear sizes: A systematic review and meta‐analysis. Orthop J Sports Med. 2023;11:23259671231180854. 37655249 10.1177/23259671231180854 PMC10467404

[6] Zhang Q , Ge H , Zhou J , Yuan C , Chen K , Cheng B . Single‐row or double‐row fixation technique for full‐thickness rotator cuff tears: A meta‐analysis. PLoS One. 2013;8:e68515. 23874649 10.1371/journal.pone.0068515 PMC3708899

[7] Imam M , Sallam A , Ernstbrunner L , et al. Three‐year functional outcome of transosseous‐equivalent double‐row vs. single‐row repair of small and large rotator cuff tears: A double‐blinded randomized controlled trial. J Shoulder Elbow Surg. 2020;29:2015‐2026. 32951642 10.1016/j.jse.2020.05.005

[8] Lapner P , Li A , Pollock JW , et al. A multicenter randomized controlled trial comparing single‐row with double‐row fixation in arthroscopic rotator cuff repair: Long‐term follow‐up. Am J Sports Med. 2021;49:3021‐3029. 34398641 10.1177/03635465211029029 PMC8411465

[9] Saridakis P , Jones G . Outcomes of single‐row and double‐row arthroscopic rotator cuff repair: A systematic review. J Bone Joint Surg Am. 2010;92:732‐742. 20194334 10.2106/JBJS.I.01295

[10] Hantes ME , Ono Y , Raoulis VA , et al. Arthroscopic single‐row versus double‐row suture bridge technique for rotator cuff tears in patients younger than 55 years: A prospective comparative study. Am J Sports Med. 2018;46:116‐121. 28942685 10.1177/0363546517728718

[11] Franceschi F , Papalia R , Franceschetti E , et al. Double‐row repair lowers the retear risk after accelerated rehabilitation. Am J Sports Med. 2016;44:948‐956. 26797698 10.1177/0363546515623031

[12] Chernchujit B , Mendoza CJP , Samsuya KKM . Blended suture‐bridge technique for arthroscopic rotator cuff repair. Arthrosc Tech. 2023;12:e569‐e574. 37138682 10.1016/j.eats.2022.12.011 PMC10150159

[13] Hernigou P , Flouzat Lachaniette CH , Delambre J , et al. Biologic augmentation of rotator cuff repair with mesenchymal stem cells during arthroscopy improves healing and prevents further tears: A case‐controlled study. Int Orthop. 2014;38:1811‐1818. 24913770 10.1007/s00264-014-2391-1

[14] Uyeki CL , Ford BT , Shuman ME , Hawthorne BC , Wellington IJ , Mazzocca AD . Biologic augmentation of rotator cuff repair: Current concepts review. Orthopedics. 2024;47:e282‐e286. 39495158 10.3928/01477447-20241028-01

[15] Mannava S , Jinnah AH , Plate JF , Stone AV , Tuohy CJ , Freehill MT . Basic shoulder arthroscopy: Beach chair patient positioning. Arthrosc Tech. 2016;5:e731‐e735. 27709029 10.1016/j.eats.2016.02.038 PMC5040097

[16] Jeong JY , Min SK , Park KM , Park YB , Han KJ , Yoo JC . Location of rotator cuff tear initiation: A magnetic resonance imaging study of 191 shoulders. Am J Sports Med. 2018;46:649‐655. 29314867 10.1177/0363546517748925

[17] Zhao J , Luo M , Pan J , et al. Risk factors affecting rotator cuff retear after arthroscopic repair: A meta‐analysis and systematic review. J Shoulder Elbow Surg. 2021;30:2660‐2670. 34089878 10.1016/j.jse.2021.05.010

[18] Mall NA , Tanaka MJ , Choi LS , Paletta GA. Factors affecting rotator cuff healing. J Bone Joint Surg Am. 2014;96:778‐788. 24806015 10.2106/JBJS.M.00583

[19] Longo UG , Carnevale A , Piergentili I , et al. Retear rates after rotator cuff surgery: A systematic review and meta‐analysis. BMC Musculoskelet Disord. 2021;22:749. 34465332 10.1186/s12891-021-04634-6 PMC8408924

[20] Wall LB , Keener JD , Brophy RH . Double‐row vs single‐row rotator cuff repair: A review of the biomechanical evidence. J Shoulder Elbow Surg. 2009;18:933‐941. 19833290 10.1016/j.jse.2009.07.002

[21] Mazzocca AD , Millett PJ , Guanche CA , Santangelo SA , Arciero RA . Arthroscopic single‐row versus double‐row suture anchor rotator cuff repair. Am J Sports Med. 2005;33:1861‐1868. 16210578 10.1177/0363546505279575

[22] Brady PC , Arrigoni P , Burkhart SS . Evaluation of residual rotator cuff defects after in vivo single‐ versus double‐row rotator cuff repairs. Arthroscopy. 2006;22:1070‐1075. 17027404 10.1016/j.arthro.2006.05.007

[23] Nelson CO , Sileo MJ , Grossman MG , Serra‐Hsu F . Single‐row modified mason‐allen versus double‐row arthroscopic rotator cuff repair: A biomechanical and surface area comparison. Arthroscopy. 2008;24:941‐948. 18657744 10.1016/j.arthro.2008.03.011

[24] Go TW , Park JE , Oh S , Cho M , Jo CH . Effect of quality of repair on clinical and structural outcomes of rotator cuff repair. Am J Sports Med. 2022;50:3915‐3923. 36341899 10.1177/03635465221130759

[25] Kim DH , Elattrache NS , Tibone JE , et al. Biomechanical comparison of a single‐row versus double‐row suture anchor technique for rotator cuff repair. Am J Sports Med. 2006;34:407‐414. 16282581 10.1177/0363546505281238

